# A systematic PLS-SEM approach on assessment of indigenous knowledge in adapting to floods; A way forward to sustainable agriculture

**DOI:** 10.3389/fpls.2022.990785

**Published:** 2022-08-25

**Authors:** Muhammad Tayyab Sohail, Shaoming Chen

**Affiliations:** ^1^School of Public Administration, Xiangtan University, Xiangtan, Hunan, China; ^2^South Asia Research Center, School of Public Administration, Xiangtan University, Xiangtan, Hunan, China; ^3^International Business School, Guangzhou City University of Technology, Guangzhou, China

**Keywords:** farmer, PLS-SEM, climate change, floods, South Punjab, Pakistan

## Abstract

The present study was conducted in one of the major agriculture areas to check farmers indigenous knowledge about the impacts of floods on their farming lives, food security, sustainable development, and risk assessment. In the current study, primary data was used to analyze the situation. A semi-structured questionnaire was distributed among farmers. We have collected a cross-sectional dataset and applied the PLS-SEM dual-stage hybrid model to test the proposed hypotheses and rank the social, economic, and technological factors according to their normalized importance. Results revealed that farmers’ knowledge associated with adaption strategies, food security, risk assessment, and livelihood assets are the most significant predictors. Farmers need to have sufficient knowledge about floods, and it can help them to adopt proper measurements. A PLS-SEM dual-stage hybrid model was used to check the relationship among all variables, which showed a significant relationship among DV, IV, and control variables. PLS-SEM direct path analysis revealed that AS (b = −0.155; *p* 0.001), FS (b = 0.343; *p* 0.001), LA (b = 0.273; *p* 0.001), RA (b = 0.147; *p* 0.006), and for FKF have statistically significant values of beta, while SD (b = −0.079NS) is not significant. These results offer support to hypotheses H1 through H4 and H5 being rejected. On the other hand, age does not have any relationship with farmers’ knowledge of floods. Our study results have important policy suggestions for governments and other stakeholders to consider in order to make useful policies for the ecosystem. The study will aid in the implementation of effective monitoring and public policies to promote integrated and sustainable development, as well as how to minimize the impacts of floods on farmers’ lives and save the ecosystem and food.

## Introduction

In recent decades, more people have become aware of the effects of climate change on the entire world. Climate change is a worldwide problem that would disproportionately affect low-income countries (Rosenzweig et al., 2001; Adger et al., 2003; Thornton et al., 2014). Due to climate change, natural disasters like floods, droughts, tsunamis, storms, and tornadoes commonly occur (Abid et al., 2016; Khan et al., 2020). Patt and Schröter (2008) stated that this phenomenon (climate change) may result in shifting climatic zones, altered rainfall patterns, and rising sea levels. The frequency of floods that are mostly caused by excessive rainfall or occur from the unintended release of water storage, such as dams, snow, or tides, is one of the most often observed effects of climate change (Kavvada and Held, 2018; Samu and Kentel, 2018). Due to low incomes and limited adaptations, impoverished agricultural and rural residents in developing countries are extremely concerned about climate change (Thornton et al., 2014; Sohail et al., 2014b; Sohail et al., 2019b). Emerging economies are more vulnerable to climate hazards than industrialized ones, and Pakistan is one of the areas most affected by climate change (Ali and Erenstein, 2017). Floods and droughts are the most prevalent natural disasters that seriously endanger humanity’s economic and social well-being, especially in developing nations with little capacity for adaptation and vulnerable populations (Strömberg, 2007; Muhammad et al., 2014; Shahab et al., 2016; Mahfooz et al., 2017, 2019, 2020; Rasool et al., 2017; Lu and Sohail, 2022; Mustafa et al., 2022a). High rates of urbanization in flood-prone locations, a huge number of sub-customary buildings, changes in land use, an increase in population density, and especially global warming, have all had a substantial impact on flooding. The intensification of extreme weather events, which also contribute to global warming and present another barrier to sustainable growth, will result in food shortages and a decline in poverty. Land use, particularly management, urban planning, and the development of flood control technologies, has a substantial impact on flood damage (Luino et al., 2018). Implementing acceptable and effective adaptation measures (such as disaster risk reduction measures) into management plans and strategies can help to minimize the impacts of natural disasters (Thomalla et al., 2006; Begum et al., 2014). Pakistan is one of the countries most susceptible to natural calamities. One of the most important environmental problems caused by global climate change is flooding, as Pakistan has experienced over the past 6–7 years (Webster, 2011; Azam and Mariani, 2012; Surminski and Oramas-Dorta, 2014; Yen et al., 2017, 2021; Sohail et al., 2020; Zinda et al., 2021; Wang and Sohail, 2022). Natural disasters, such as floods, droughts, scorching temperatures, and high rates of pests and disease, have been more frequent and severe in the United States over the previous 10 years (Smit and Skinner, 2002). In 2010, 2011, 2012, and 2015, Pakistan was ranked seventh for climate change and the hazards it poses, and sixteenth for flood vulnerability (Kreft et al., 2013; Abid et al., 2016; Khan et al., 2020). The deterioration of these weather patterns will hasten global warming and introduce fresh barriers to sustainable development, increasing poverty and causing a food shortage (Sohail et al., 2017; Luino et al., 2018; Yat et al., 2018; Yasar et al., 2019; Sohail et al., 2021a; Sohail et al., 2021a; Sohail et al., 2021a). It is envisaged that developing countries can minimize natural hazards and dangers connected with extreme climate change by incorporating appropriate and effective adaptation measures (including disaster risk reduction measures) into their management plans and strategies (Hoanh et al., 2006; Thomalla et al., 2006; Atta-ur-Rahman et al., 2010; Azam and Mariani, 2012; Begum et al., 2014; Jiang et al., 2021). In the previous 10 years, the freuqency and intensity of natural disasters in the nation have increased. Examples include floods, droughts, painful heat, and high rates of pests and diseases (Smit and Skinner, 2002). According to Abid et al. (2016) and Khan et al. (2020), Pakistan was seventh among the countries most vulnerable to the risks associated with climate change, while it was ranked seventeenth most vulnerable to floods in 2010, 2011, 2012, and 2015, respectively (Kreft et al., 2013). These analyses make a strong argument for the creation of a specialized, market-based flood insurance policy as a climate change adaptation measure. It is crucial to research the factors that affect farmers’ willingness to pay for flood insurance before starting a program in order to reduce their flood risk, regardless of whether they accept it. Along with risk attitudes and subjective estimates of the risk of flooding, they also include social and geographic factors. Farmers’ flood risk behaviors, in particular their risk perception and attitude, may be crucial when considering willingness to pay for flood insurance. Their decisions about agricultural productivity, investments, and farm management may also be impacted by this (Champonnois and Erdlenbruch, 2021). At the moment, it’s thought that managing flood risk necessitates using subjective risk assessment, including risk perception (Lechowska, 2018). The farmer’s risk aversion, however, affects their economic behavior and the coping mechanisms they use to decrease the effects of numerous disaster-related threats (Saqib et al., 2016; Zhao et al., 2019, 2022a,b; Sohail et al., 2021b,d). In this study, the influence of various factors on farmers’ perceptions of floods and their potential consequences on their farming practices were assessed. Floods won’t have much of an impact on farmers’ regular activities provided they have the equipment and knowledge to handle such disasters. Based on the literature that is already available, the following questions have been raised: Global warming-related over-melting of the Karakoram glacier is expected to rise by 50% in the first half of the century before falling by 40% by the end of the century (Rees and Collins, 2006). There is significant geographical variation in the amount of precipitation and air temperature along the Indus basin tributaries that flow out of Punjab, Pakistan (Saqib et al., 2016). These spatial and temporal variations in temperature and precipitation have a direct impact on the hydrological cycles and climatic extremes in the area. Floods are the worst of all-natural disasters that hurt humans; they can cause a large number of fatalities and put populations at risk of economic and social loss (Ali et al., 2013). Data from the past shows that floods in Pakistan have increased in frequency, size, and duration during the past few decades. The flood history of Pakistan from 1950 to 2019 is presented in Table 1. However, the floods that hit Pakistan in 2010, 2011, 2012, and 2014 are typical examples that significantly impacted both the GDP and the lives of the populace (Mustafa and Wescoat, 1997; Abid et al., 2016). Future forecasts of potentially catastrophic events like earthquakes, floods, and droughts are important to estimate the losses caused by natural disasters like earthquakes. In this study, we examined the impending floods in Pakistan and their consequences on GDP, impacted populations, and urban damage. A detailed literature review was conducted to find out more about Pakistan’s vulnerability to floods and previous flood disasters. To assess the potential harm that flooding could cause, look into the prospective effects on GDP, and illustrate the long-term effects, various flood prevention measures were used based on the literature research. These are some main research questions on the basis of literature. What are the influences of farmers’ knowledge about floods to minimize the impacts of natural disasters (floods) on their daily lives? What is the stabilized importance of adaptation strategies against floods in South Punjab, Pakistan? Does gender play a moderating role in farmers’ knowledge and adaptation strategies against floods? The main purposes of this study are (1) to analyze the influences of farmers’ knowledge about floods to minimize the impacts of farmers on their daily lives and food security livelihood assets; (2) to determine the possible causes of floods and estimate future flood-related risks in the country; (3) Farmers’ views about floods and adaption strategies against them, and (4) How can we minimize the impacts of floods on farmers’ lives and safe ecosystems and foods?

**TABLE 1:** Flood in Pakistan (1950–2019).

Sr. No.	Period	Floods (Numbers)	Economics (Loss the US $)	Population (Affected)	Deaths (Numbers)
1	2010–2019	30	18113000	36495066	4,713
2	2000–2009	33	706148	9574150	2,265
3	1990–1999	14	1092230	18148606	4,180
4	1980–1989	7	1367000	1304900	519
5	1970–1979	5	1166500	13637200	2,066
6	1960–1969	2	3300	224427	32
7	1950–1959	6	1719000	36954	3,691

Khan et al. (2021).

**FIGURE 1:**
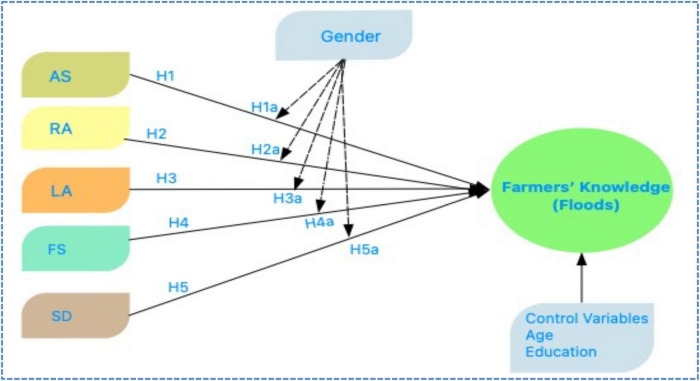
Research model.

To expand, preserve self-sufficiency, and guarantee access to enough food, the agricultural sector needs to implement effective climate change adaptation measures, particularly in the wake of natural disasters like floods (Ferdushi et al., 2019). Sharing information/knowledge and understanding about adaptation is one of these and is crucial for boosting the agricultural sector’s resilience in Bangladesh since it will help protect farmers’ livelihoods and agricultural sustainability (Budhathoki et al., 2020). In this regard, smallholder farmers who are already feeling the effects of climate change have begun to apply a range of agricultural approaches (Alam et al., 2017; Manoj, 2017). The opinions, knowledge, and awareness of climate change among farmers are necessary for adaptation strategies. Therefore, farmers need to have a thorough awareness of climate change to use the most effective adaptation strategies and methods (Somda et al., 2017). One of the best and most important risk management techniques for natural catastrophes is the employment of dependable strategies. It has also been suggested as one of the adaptation strategies for climate change (Goodwin and Smith, 1995; Goodwin and Mahul, 2004). Farmers that are aware of natural disasters can utilize good management to prevent them (Di Falco et al., 2014; Jian et al., 2021; Sohail et al., 2021a,Sohail et al., 2022a; Lan et al., 2022; Li et al., 2022a,b). Over the years, crop insurance has been employed in numerous ways and for numerous purposes in numerous countries, with variable degrees of success (Vandeveer, 2001; Enjolras et al., 2012; Parvin et al., 2016; Mustafa et al., 2021). Food security depends on agriculture and farmers managing natural disasters effectively and using the right techniques to minimize their effects on food production (Yang et al., 2015). Due to the increased frequency of natural disasters like droughts and flooding, global warming harms agricultural production (Gong et al., 2003; Sun et al., 2005; Zhao et al., 2009; Zhang et al., 2011). Due to the climate, some farmers, particularly smallholder farmers, are vulnerable and lose their livelihoods (Sen, 1981; Valdivia et al., 2010). Climate change exacerbates global inequality because those most affected are also the least to blame for it (Barnett, 2006; Roberts and Parks, 2007). Vulnerable households employ their limited resources to deal with a changing range of stressors, such as social, economic, political, and environmental stress (Mortimore and Adams, 2001; Sohail et al., 2014a; Sohail et al., 2015; Sohail et al., 2019a; Liu N. et al., 2022; Liu Y. et al., 2022; Sohail et al., 2022a; Sohail et al., 2022a). It is believed that natural disasters are mostly to blame for the recent increases in undernourishment and food insecurity around the world, particularly in developing countries where farmers’ livelihoods are more exposed to and vulnerable to calamities brought on by climate change. Furthermore, it is widely established that natural catastrophes reduce smallholder farmers’ resistance to threats, shocks, and pressures while increasing the vulnerability of their livelihoods. Despite being a worry on a global scale, the implications of human-caused climate change vary depending on the location, system, family, and community. Because of this, the susceptibility of each object varies (Adger and Kelly, 1999; Adger et al., 2004, 2006; Zahid and Khurshid, 2018; Zahid et al., 2022a,b). This has led some researchers to advocate using local assessments to gauge a region’s vulnerability to climate change (Deressa et al., 2009; Below et al., 2012). Understanding the vulnerability of rural households’ livelihoods is essential to establishing adaptation strategies and practical solutions/policies for decreasing climate-related risks and strengthening their resilience, particularly in countries that are heavily dependent on agriculture. By encouraging landowners to invest in long-term soil remediation, land tenure rights are thought to improve sustainable natural resource management and ensure the livelihoods of nearby households (Jakobsen et al., 2007). This investigation led us to develop the following research proposal: H1: Adaption strategies (AS) against floods are positively associated with farmers’ knowledge of floods. H2: The Risk Assessment (RA) against floods is positively associated with farmers’ knowledge of floods. H3: Livelihood Assets (LA) of farmers (which help) against floods are positively associated with the farmer’s knowledge of floods. H4: Impacts of floods on food production. Food security (FS) against floods is positively associated with farmers’ knowledge (floods). H5: Floods’ impacts on the sustainable development (SD) of a specific area are positively associated with farmers’ knowledge (floods). The explanatory variables are linked by age, gender, and educational attainment as significant modiators (Mustafa et al., 2022a,b, c). Men and women approach decision-making with very diverse perspectives on the surrounding environment. There are numerous ways in which the world appears extremely different when viewed through the eyes of a man as opposed to a woman. According to Pakistani studies (Mustafa et al., 2022d), environmental knowledge has a significant impact on men’s intentions but has a minimal effect on women’s. Mustafa, Tengyue, Jamil, et al. argue that there are differences in the way that the relationships between different factors hold across sexes (2022e). Because of the observed pattern of gender differences, we can predict that male and female farmers will have quite different preferences for adapting to their environment. As a result, it is assumed there is a strong relationship between farmers’ knowledge of AS, RA, LA, FS, and SD (H1a-H5a), all of which are strongly affected by their gender (floods) Figure 1.

## Materials and methods

### Study area

**FIGURE 2:**
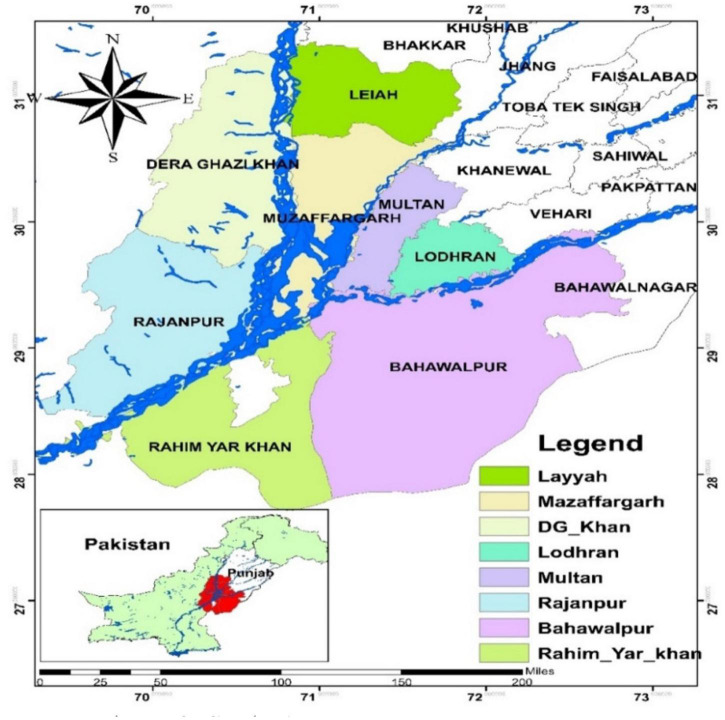
Study area.

Punjab is the most populated area with five rivers. Most of the people from this province work in farming. The Khyber Pukhtunkhwa Province and the federal capital region of Islamabad are located north of Punjab. The Indian Punjab and Rajasthan are to the south-east, Sindh is to the south-west, Baluchistan and the Federally Administered Tribal Areas are to the west, and Azad Kashmir is to the north-east (FATA). Although the province is predominantly flat, the extreme north and south-west contain some hilly areas. In addition, Cholistan is a desert belt in the south-eastern region, and the Potohar plateau is a plateau adjacent to the mountains. This province is traversed by all five of the country’s major rivers: the Indus, Jhelum, Chenab, and Ravi, in addition to the Sutlej. They originate in the Himalayas and travel from north to south. They are primitive, and during monsoon rains in the summer, the water level rises, causing occasional flooding. Punjab is the province with the largest population in Pakistan. As of the 1998 Census, the province had a population of 7,258,500 people. It includes Lahore, Faisalabad, Rawalpindi, Multan, and Gujranwala, which are some of the most populous cities in the country (Figure 2).

### Data sources and data preparation

This study was carried out in Punjab province, based on farmers’ experiences with flooding, climate change, and adaption measures (Sohail et al., 2021a,2022a). This area of Pakistan is primarily agricultural, making it particularly susceptible to natural disasters, including riverbank erosion, floods, and droughts. Floods are mostly caused by the Indus rivers, making this area highly vulnerable to natural disasters. A systematic questionnaire was used to conduct in-depth interviews with farm households that had experienced flooding to gather primary data (Figure 2). To analyze the relationship between farmers’ knowledge about floods to minimize their impacts on their daily lives and to determine the possible causes of floods; estimate future flood-related risks in the country; famers’ views about floods and adaption strategies against them; and how to minimize impacts of floods on farmers’ lives and safe ecosystems and foods. Eight districts in the Punjab province were selected based on the degree of previous flood damage, flooding history, vulnerability, and agricultural significance. A semi-structured questionnaire adapted from a previous study was used to gather information from eight districts in South Punjab, Pakistan (Sohail, 2013; Sohail et al., 2013; Fahad and Wang, 2020; Sohail et al., 2022a). To gather information from farmers in South Punjab, Pakistan. We informed farmers about climate change as well as information about the study’s objective. Data was entered into SPSS 25 and Smart-PLS for further analysis after data collection. The information was examined using statistical techniques. Because PLS-SEM is one of the most efficient methods for predicting outcomes, PLS-SEM was used to analyze the data in our study to check the relationship between DV and IV variables. PLS-SEM is the method that is most often recommended for predicting and evaluating explained variables in order to account for the most possible differences.

## Results and discussion

For this study, 1,200 farmers from Pakistan’s Punjab Province were interviewed. Table 1 includes numerous internal variables that may influence a farmer’s response and adaptability, including personal characteristics, unique conditions, and farming practices (Bryan et al., 2013). Deressa et al. (2009) found that a farmer’s awareness is positively associated with their farming background and education. Existing research indicates that farmers’ perceptions of climate change and its implications are influenced by the features of farmland and farmers’ demographic assets (Singh et al., 2017). Previous research supports the importance of farmers’ socioeconomic and demographic characteristics in the implementation of climate change adaptation techniques on their farms.

**FIGURE 3:**
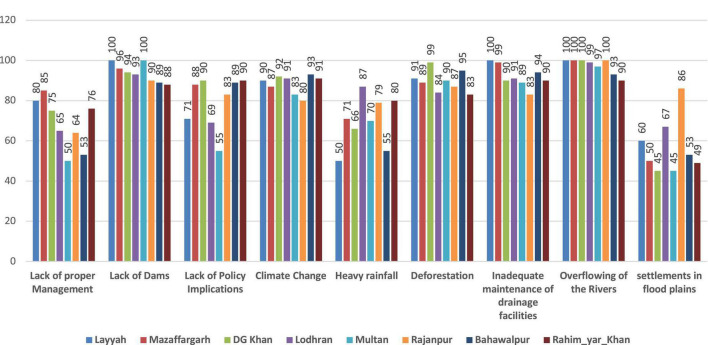
Main reasons for overflooding in South Punjab Pakistan (%).

Figure 3 elaborates the main reasons for the floods in South Punjab, Pakistan. Without a doubt, the effects of climate change have become more apparent over the last few decades (Patt and Schröter, 2008). The main indicators which were encompassed to check perception were lack of proper management, lack of dams, lack of policy implications, climate change, heavy rainfall, deforestation, inadequate maintenance of drainage facilities, and overflowing of rivers and settlements in flood plains. According to farmer responses about a lack of proper management in Layyah 80%, Muzaffargarh 85%, DG Khan 75%, Lodhran 65%, Multan 50%, Rajanpur 64%, Bahawalpur 53%, and Rahim_Yar_Khan 76%, respectively, flooding could be the main threat to farmers in that area due to the Indus River provided sufficient evidence of the impacts of temperature stress and floods on farmers’ lives (Maheen and Hoban, 2017). Farmers in Layyah responded 71%, Muzaffargarh 88%, DG Khan 90%, Lodhran 69%, Multan 55%, Rajanpur 83%, Bahawalpur 89%, and Rahim_Yar_Khan 90% to a lack of policy implications. Both short-term and long-term actions are essential to effectively managing the losses caused by floods. When undertaking rational decision-and risk analysis regarding catastrophic events, the gathering of information is a key stage. Making more informed decisions can be aided by the capacity to draw from a sizable body of knowledge from prior decisions and safety measures put in place in disaster zones. With a solid conceptual framework, it can be simpler to understand perspectives on fair procedures and results. A standardized model makes data collection simpler and can aid in the development of a knowledge base that various model instances can use. This article explores the trends in national flood control as well as flooding in the major basins. Flooding in the Indus Basin is mostly brought on by monsoon precipitation, whereas flooding in the Kharan Basin and Makran Coastal Area is brought on by Mediterranean waves and cyclones that occur over the Arabian Sea. Floods in the Indus Basin have caused substantial financial harm. Since Pakistan’s founding in 1947, the government has allotted a sizeable percentage of its budget toward relief efforts and flood relief projects. Several provincial and federal laws, regulations, agreements, and treaties influence the nation’s flood policy. The institutional setting for flood hazard and disaster management has evolved. However, data does not show a material drop in the flood-to-damage ratio. Examining and maximizing the interactions between structural and non-structural measures is necessary for more effective flood management. All rivers in Pakistan’s transboundary rivers flow *via* India, and upstream activities mostly determine the shape of a flood wave’s shape. Layyah, Muzaffargarh, DG Khan, Lodhran, Multan, Rajanpur, Bahawalpur, and Rahim Yar Khan all received 100% farmer feedback on the lack of a dam. Farmers in Layyah, Muzaffargarh, DG Khan, Lodhran, Multan, Rajanpur, Bahawalpur, and Rahim Yar Khan are concerned about climate change. Recent environmental degradation in Pakistan caused floods in 2010 and 2011. In the future, more disasters are expected. Changing environmental conditions like urbanization, population increase, etc., threaten Pakistan’s ecosystem and biodiversity. According to the Federal Bureau of Statistics, the forest area declined by 3% between 2000 and 2005. Overgrazing, farming methods, and rural wood consumption create this. Northern Pakistan’s rising temperatures are due to deforestation. In the past 50 years, both the risky area’s population and human settlement vulnerability have increased. Human choices and investments enhance catastrophic losses (Benson and Clay, 2004). According to Jamshed et al. (2019), Pakistan’s socioeconomic basis has been undermined by flooding. The country is one of the most flood-prone in the world, with frequency and intensity rising (Hirabayashi et al., 2013). The 2010 floods alone inundated 2.1 million acres of standing crops and harmed nearly 400,000 animals. Khyber-Pakhtunkhwa (KP) remains highly vulnerable to flooding and its catastrophic impacts. Pakistan is a growing nation attempting to advance in many areas, but a large portion of the population, especially farmers in rural disaster zones, lives in poor conditions (Fahad et al., 2020). In 2010, 2011, and 2014, Pakistan witnessed devastating floods in several places that devastated forestry, cattle, fisheries, infrastructure, fertilizers, animal barns, and more than 250,000 farmhouses on 1 million acres of cultivated land (NDMA, 2014). Crop failure, low yields, and livestock fatalities can result from these disasters (Harvey, 2014).

**FIGURE 4:**
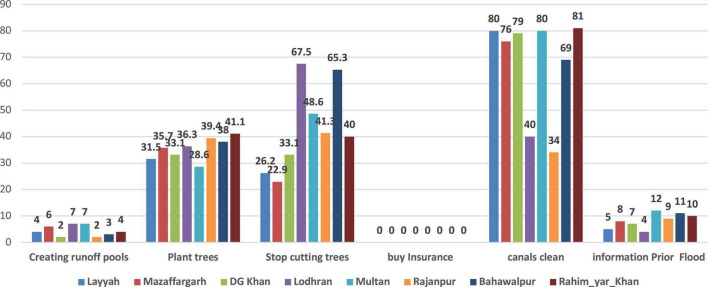
Adaption strategies/measures (%) against floods by farmers in South Punjab Pakistan.

The present study also included adaptation techniques against floods; these parameters were: creating runoff pools; stopping cutting trees; planting trees; buying insurance; canal cleaning; and information before floods (Figure 4). It is very common for many farmers to choose different techniques to deal with livelihood risks (Kuang et al., 2020). It is reported that similar kinds of adaptation measures like changing planting trees, cleaning canals with the government, and other techniques are adopted by farmers in Pakistan (Gorst et al., 2015). According to research, small farmers are more likely than large farms to have problems responding to climate change (Jamshidi et al., 2019). Additionally, the effect of climatic threats on farmers’ income, family food, and security was even more pronounced for farmers with limited access to resources (Shukla et al., 2019). Researchers discovered that the first step in responding to climate change was to modify how people thought about it in another study conducted in Africa (Trinh et al., 2018). Farmers must be aware of the threats posed by the climate and how to adapt to them. They can do this to boost agricultural productivity and reduce risk (Fahad and Wang, 2020). The physical, natural, and social resources of farmers aid in the adoption of adaptation techniques (Kuang et al., 2020). The percentage of farmers who claimed they wished to create runoff pools was 4% in Layyah, 6% in Muzaffargarh, 2% in DG Khan, 93% in Lodhran, 100% in Multan, 90% in Rajanpur, 89.9% in Bahawalpur, and 88% in Rahim-Yar-Khan. City life can be severely disrupted by runoff flooding. As a result, runoff flooding should not be a problem for a city. The study of ecology gave rise to the concept of resilience. A system’s resilience is its capacity to handle external changes while maintaining its identity, structure, and functions (Holling, 1973). A system that has resilience can adjust to stress and become less vulnerable. The socio-ecological system is utilized in conjunction with the emerging concept of ecological resilience as a comprehensive human-in-nature perspective (Berkes and Folke, 1998). Dam failure disasters are a major concern around the world, especially in poorer nations where dam safety hasn’t received much attention (WB, 1990; Dam, 2011). Numerous organizations from around the world are looking for tools and strategies to improve the situation, such as increased data collection, performance measurement, and rankings, as a result of the development of water infrastructure in developing countries and the deterioration and poor management of older infrastructure (Berg). (Bryan et al., 2009). 31.5 percent of farmers in Layyah said they would plant trees; 35.7 percent in Muzaffargarh; 33.1 percent in DG Khan; 28.6 percent in Lodhran; 39.4 percent in Multan; 41.1 percent in Rajanpur; and 41.1 percent in Rahim-Yar-Khan. There is some evidence that, at least occasionally, clearing a landscape of trees increases the likelihood of flooding (Bruijnzeel, 1990, 2004). According to the postulated process, less vegetation is thought to increase runoff because less rainfall is captured and less water evaporates from the tree canopy. This makes it more difficult for water to seep into the soil, which is associated with a decrease in the hydraulic conductivity (infiltration rate) of soils (Clark et al., 2021). So, the idea that the loss of natural habitat increases the danger and severity of catastrophic floods, as well as the harm they bring to people and their property, has arisen as a result of the rapid rate at which forests are currently being chopped down (Achard et al., 2002; Laurance, 2004; Clark et al., 2021). According to what farmers in Layyah, Muzaffargarh, DG Khan, Lodhran, Bahawalpur, and Rahim Yar Khan said regarding not cutting down trees, 26.2 percent, 22.9 percent, 33.1 percent, 33.1 percent, 67.5 percent, 48.6 percent, 41.3 percent, 65.5 percent, and 40 percent, respectively, said they would refrain from doing so. According to farmer opinions on purchasing insurance against natural catastrophes, 0% of farmers in Layyah, 0% in Muzaffargarh, 0% in DG Khan, 0% in Lodhran, 0% in Multan, 0% in Rajanpur, 0% in Bahawalpur, and 0% in Rahim-Yar-Khan purchased insurance. One of the most significant methods of risk transmission is insurance. By eliminating or lowering the financial risks, it can assist in managing the flood risk before it occurs (Surminski and Oramas-Dorta, 2013). Although this kind of policy is uncommon in underdeveloped nations, many industrialized nations use flood insurance as a non-structural method of dealing with flooding (Champonnois and Erdlenbruch, 2021). Even if structural adjustments can prevent actual property damage and fatalities, flood insurance can significantly reduce economic losses, especially in low-income nations that are prone to floods (Aliagha et al., 2014). People have observed that small and medium-sized farmers have had an extremely difficult time recovering the money they lost due to the floods (Wedawatta and Ingirige, 2012). Small farmers in Bangladesh may therefore find flood insurance to be a wise investment because it enables them to adapt to climate change by covering insured losses in the event of a flood disaster. According to Hossain et al. (2019), climate-related floods, heavy rain, drought, cyclones, and storms have a severe impact on agricultural output as well as on buildings, dams, and other architectural structures in underdeveloped developing nations. The impact on rural income and food security is significant. As a result, the poorest nations in South and Southeast Asia will be severely harmed (Vinke et al., 2017). Extreme weather events have increased in frequency and severity during the past few decades (Hossain et al., 2019). One of the most dangerous natural calamities that individuals might experience is flooding. They might result in numerous fatalities as well as social and economic issues (Ali et al., 2013; Mondal et al., 2020).

### PLS-SEM and multivariate assumptions

One of the most effective methods for predicting outcomes and explanatory variables is PLS-SEM to examine relationships between variables (Hair et al., 2020). PLS-SEM generates more accurate results with smaller sample sizes. Internal and external processing of every model is possible simultaneously. Complex route models can be studied using this data collection (Hair et al., 2021; Mustafa and Wen, 2022; Mustafa et al., 2022e). The non-linear account interactions in the model necessitate a two-stage analysis. A PLS-based route modeling method is verified twice to ensure accuracy and dependability. Analyze the validity, reliability, and convergent validity of a structural model before developing an inner model or link between latent components. According to academics, multivariate assumptions need to be verified before an investigation (Mustafa and Wen, 2022; Mustafa et al., 2022e). Data assumptions include linearity, multicollinearity, and homoscedasticity. A Kolmogorov-Smirnov analysis was used to determine whether the data set was normal. There are both linear and non-linear interactions between the explanatory variables and the exploratory variables. Finally, we examined the VIF of the model for collinearity. In Table 2, all variables have VIF values under 5. When VIF is less than 5, according to them, dataset collinearity isn’t a concern.

**TABLE 2:** Demographic information of participants (%).

		Layyah	Muzaffargarh	DG Khan	Lodhran	Multan	Rajanpur	Bahawalpur	Rahim_yar_ Khan
Gender	Male	85.4	81.4	73.8	75	77.1	71.6	74	72.2
	Female	14.6	18.6	26.2	25	22.9	28.4	26	22.8
Age (Years)	18–25	17.7	13.6	13.8	15	19.3	23.2	22.7	20.6
	26–35	37.7	20.7	37.2	33.1	32.9	34.8	33.3	26.7
	36–45	28.5	43.6	26.2	40.6	32.1	28.4	32.7	41.1
	Above 45	16.2	22.1	22.8	11.3	15.7	13.5	11.3	11.7
Education (Years)	Primary School	31.1	23.6	25.5	45.6	52.1	49	39.3	30.6
	High School	43.1	50	54.5	40.6	39.3	41.3	34.7	38.9
	College Level	14.6	15.7	11	9.4	5	7.7	18.7	21.1
	University level	9.2	10.7	9	4.4	3.6	1.9	7.3	9.4
Farming present land (Years)	1–5	26.2	35	46.2	45	53.6	49	54	61.7
	6–10	50	32.9	28.3	31.9	20.7	36.1	28.7	28.9
	11–15	10	21.4	18.6	15.6	22.1	9	12.7	5.6
	16-above	13.8	10.7	6.9	7.5	3.6	5.8	4.7	3.9
Plowing per year	Once	16.9	3.6	4.1	6.9	8.6	5.8	8	5
	Twice	70	78.6	80.7	61.3	63.6	56.8	45.3	45
	Three-more	31.1	17.9	15.2	31.9	27.9	37.4	46.7	50

**FIGURE 5:**
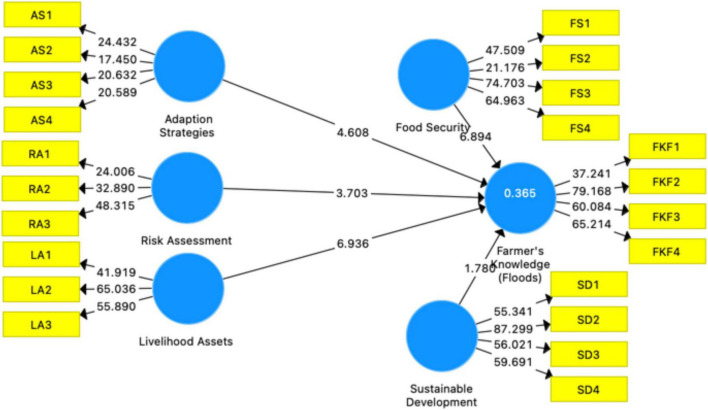
Measurement model.

Convergent and discriminant validity should be examined when evaluating measurement models. To determine if the concept indicators accurately assessed the research variables, we evaluated the instrument’s dependability using item loading and Cronbach’s alpha. The average variance extracted (AVE) and composite reliability (CR) show how much the hidden construct offsets indicator variance. The reliability of each item is assessed using factor loadings on linked structures (Figure 3 and Table 2). For a component to be deemed significant, its outer loading must be at least 0.6 (Hair et al., 2020). To boost confidence, Cronbach’s alpha should be higher than or close to 0.7 for all conditions. According to Werts et al. (1974), this improves reliability. Composite reliability (CR), as well as Cronbach’s alpha, were measured. The conventional method was replaced by this (Werts et al., 1974). Strong dependability ratings (>0.7) support these conclusions, and convergent validity estimates above 0.50 are shown in Table 2 (Hair et al., 2020). Figure 5 displays computations of the PLS algorithmic measurements model for all variables and DV/IV variables (Figure 5).

The discriminant validity of the proposed model is evaluated using the Fornell-Larcker criterion and heterotrait-monotrait (HTMT) ratios (Hair et al., 2020, Hair et al., 2021). The strongest significant correlation of variables in each column in Table 3 demonstrates that the Fornell-Larcker criteria have been utilized to demonstrate discriminant validity (Fornell and Larcker, 1981; Henseler et al., 2015). The values of the Fornell-Larcker criterion, the standard deviation, and the mean of all the variables are shown in Table 3. Even if it was adequate for measuring discriminant validity, the Fornell-Larcker criteria couldn’t distinguish between its lack and presence, they said. This led to the use of the HTMT to test discriminant validity. Table 4 shows the HTMT values for each of the research criteria. As per requirement, values of HTMT must be below 0.90 to prove data validity, and in this research, HTMT values were below 0.90, which showed this research data was valid (Henseler, Ringle, Sarstedt) (Table 4).

**TABLE 3:** Validity and reliability analysis.

Variables	Items	Loadings	VIF	T Statistics	α	CR	AVE
*Adaption Strategies*	AS1	0.778***	1.511	24.354	0.740	0.753	0.555
	AS2	0.721***	1.555	17.598			
	AS3	0.743***	1.520	20.741			
	AS4	0.736***	1.511	21.012			
*Risk Assessment*	RA1	0.749***	1.315	24.599	0.880	0.833	0.736
	RA2	0.803***	1.530	32.982			
	RA3	0.852***	1.511	48.756			
*Food Security*	FS1	0.830***	1.726	46.442	0.847	0.874	0.686
	FS2	0.696***	1.479	21.405			
	FS3	0.891***	2.873	74.845			
	FS4	0.882***	2.897	64.785			
*Livelihood Assets*	LA1	0.846***	1.844	41.425	0.829	0.831	0745
	LA2	0.880***	2.092	65.747			
	LA3	0.863***	1.826	56.130			
*Sustainable Development*	SD1	0.854^**^	2.285	55.681	0.725	0.743	0.644
	SD2	0.892**	2.538	88.514			
	SD3	0.857**	2.380	56.166			
	SD4	0.868**	2.438	58.923			
*Farmer’s Knowledge (Floods)*	FKF1	0.811***	1.870	37.581	0.891	0.901	0.753
	FKF2	0.885***	2.616	81.021			
	FKF3	0.866***	2.300	59.459			
	FKF4	0.869***	2.372	66.316			

α > 0.7; CR > 0.7; AVE > 0.5; VIF < 5; ***Significant at p < 0.001.

**TABLE 4:** Fornell–Larcker criterion.

	*STDEV*	*Mean*	*AS*	*FKF*	*FS*	*LA*	*RA*	*SD*
*AS*	0.024	0.551	**0.745**					
*FKF*	0.019	0,735	0.341	**0.858**				
*FS*	0.016	0.685	0.225	0.436	**0.828**			
*LA*	0.019	0.744	0.313	0.473	0.297	**0.863**		
*RA*	0.019	0.642	0.366	0.419	0.347	0.446	**0.803**	
*SD*	0.015	0.752	0.332	0.324	0.660	0.320	0.380	**0.868**

Bold diagonal values are the square root of AVE. AS, adaption strategies; FKF, farmer’s knowledge (floods); FS, food security; LA, livelihood Assets; RA, risk assessment; SD, sustainable development: STDEV, standard deviation.

**FIGURE 6:**
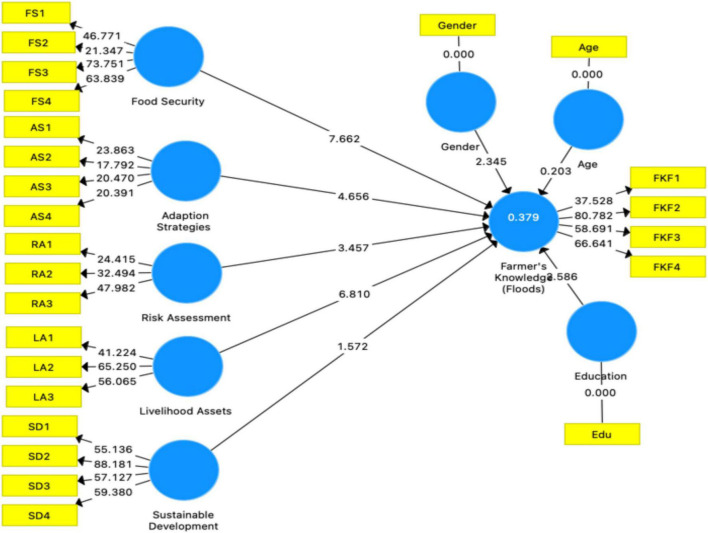
PLS-SEM path model.

Structural model research is the second phase of PLS-SEM evaluation. Examine the predictive relevance, multicollinearity, empirical significance, and confidence of the structural path model. Evaluation of the structural route model’s dependability. This study used data guidelines from Hair et al. (2021) to analyze the structural model. To determine how various factors impact FKF (Figure 6 and Table 5) elaborate on the real situation of structural model research. As per the beta value and significance of this study, H1-H4 were significant and showed a relationship with IV, while H5 was insignificant. Under this research, H1-H4 hypotheses were approved while H5 was rejected. R2 and adjusted R2 are 0.379 and 0.372, respectively Table 6. This study found that control variables (gender and education) showed a significant direct relationship with DV. This study found that farmers’ awareness of flooding and their capacity to cope with natural disasters are influenced by their gender and education (Table 5). To test the validity of the hypotheses that had been put forward previously, we began by examining the causal relationships that were already known to exist between the different variables. Following that, we carried out a bootstrapping test using 5,000 replicates to evaluate the degree to which our findings were consistent with the hypothesis (Mustafa and Wen, 2022; Mustafa et al., 2022e). PLS-SEM direct path analysis rveealed that AS (b = −0.155; *p* 0.001), FS (b = 0.343; *p* 0.001), LA (b = 0.273; *p* 0.001), RA (b = 0.147; *p* 0.006), and for FKF have statistically significant values of beta, while SD (b = −0.079NS) is not significant. These results offer support to hypotheses H1 through H4 and H5 being rejected. We have also looked at the levels of education and gender of the respondents as control variables have a significant relationship with the dependent variable. On the other hand, age does not have any relationship with farmers’ knowledge of floods (Figure 6 and Table 5).

**TABLE 5:** HTMT ratio.

*Variables*	*AS*	*FKF*	*FS*	*LA*	*RA*	*SD*
***AS***						
***FKF***	0.401					
***FS***	0.260	0.492				
***LA***	0.390	0.552	0.344			
***RA***	0.480	0.519	0.445	0.569		
***SD***	0.387	0.361	0.767	0.369	0.468	

AS, adaption strategies; FKF, farmer’s knowledge (floods); FS, food security; LA, livelihood assets; RA, risk assessment; SD, sustainable development.

**TABLE 6:** Path analysis (PLS-SEM).

*Statistical Paths*	*Beta* (β)	*Std. Dev*	*T-Value*
*AS -* > *FKF*	0.155***	0.033	4.655
*FS -* > *FKF*	0.343***	0.044	7.662
*LA -* > *FKF*	0.273***	0.040	6.809
*RA -* > *FKF*	0.147***	0.042	3.457
*SD -* > *FKF*	−0.079^NS^	0.050	1.572
***Control Variables***			
*Age -* > *FKF*	−0.000^NS^	0.029	0.202
*EDU -* > *FKF*	0.074***	0.028	2.586
*Gender-* > *FKF*	0.078***	0.033	2.345
*R* ^2^	0.379		
*Adjusted R* ^2^	0.372		

***Significant at p < 0.001, NS, not supported; AS, adaption strategies; FKF, farmer’s knowledge (floods); FS, food security; LA, livelihood assets; RA, risk assessment; SD, sustainable development; STDEV, standard deviation.

## Conclusion

This research was carried out in one of the important agricultural regions of Pakistan to check farmers’ knowledge about the impacts of floods on their farming lives, food security, sustainable development, and risk assessment. Floods are very common in this study area, which has very adverse effects on farmers’ lives and food security. Flood types vary considerably across the country because of differences in physiographic, climatic, hydrologic, demographic, and socioeconomic features. A PLS-SEM dual-stage hybrid model was used to check the relationship among all variables, which showed a significant relationship among DV, IV, and control variables. Following that, we carried out a bootstrapping test using 5,000 replicates to evaluate the degree to which our findings were consistent with the hypothesis. PLS-SEM direct path analysis revealed that AS, FS, LA, RA, and FKF had statistically significant beta () while SD was not significant. These results offer support to hypotheses H1 through H4 and H5 being rejected. As control variables, we also looked at how much education the respondents had and what gender they were. Both of these things have a strong relationship with the dependent variable. On the other hand, age does not have any relationship with farmers’ knowledge of floods. This study can help policymakers and government officials to make suitable policies for this study area, and it can help farmers to get more knowledge about floods.

## Data availability statement

The original contributions presented in the study are included in the article/supplementary material, further inquiries can be directed to the corresponding author.

## Ethics statement

Ethics review and approval/written informed consent was not required as per local legislation and institutional requirements.

## Author contributions

MS: conceptualization, methodology, software, and writing–original draft, supervision and final draft approval, data collection and analyzing, editing, and data collection. SC: visualization and investigation. Both authors contributed to the article and approved the submitted version.
